# Research on Performance of SBS-PPA and SBR-PPA Compound Modified Asphalts

**DOI:** 10.3390/ma15062112

**Published:** 2022-03-13

**Authors:** Jianguo Wei, Song Shi, Yuming Zhou, Zhiyuan Chen, Fan Yu, Zhuyi Peng, Xurui Duan

**Affiliations:** 1 School of Traffic and Transportation Engineering, Changsha University of Science and Technology, Changsha 410004, China; jianguowei9969@126.com (J.W.); songs20222022@163.com (S.S.); chenzycsust@163.com (Z.C.); 15673102532@163.com (F.Y.); p19375175428@163.com (Z.P.); duanxuruile@163.com (X.D.); 2 Henan Railway Construction & Investment Group Co., Ltd., Zhengzhou 450018, China; 3 National Engineering Laboratory of Highway Maintenance Technology, Changsha University of Science and Technology, Changsha 410004, China

**Keywords:** road engineering, polyphosphoric acid, dynamic shear rheological test, temperature sensing performance, aging performance

## Abstract

Although several studies indicated that the addition of Styrene-Butadiene-Styrene (SBS) and Styrene-Butadiene Rubber (SBR) bring a lot of benefits on properties of asphalt binders, high production costs and poor storage stability confine the manufacture of better modified asphalt. To reduce the production costs, polyphosphoric acid (PPA) was applied to prepare better compound modified asphalt binders. In this research, five PPA (0.5%, 0.75%, 1.0%, 1.25% and 1.5%) and two SBR/SBS (4% and 6%) concentrations were selected. Dynamic shear rheometer (DSR) and Bending Beam Rheometer (BBR) tests were performed to evaluate the rheological properties of the compound modified asphalt. Rolling Thin Film Oven (RTFO) test was performed to evaluate the aging properties of the compound modified asphalts. The results indicate that SBS/SBR modified asphalts with the addition of PPA show better high-temperature properties significantly, the ability of asphalt to resist rutting is improved, and the elastic recovery is increased. However, the low-temperature properties of the compound modified asphalts are degraded by increasing the creep stiffness (S) and decreasing the creep rate (m). At the same time, RTFO tests results show that PPA was less prone to oxidation to improve the anti-aging ability of modified asphalts. Overall, the combination of 4% SBS and 0.75–1.0% PPA, the combination of 4% SBR and 0.5–0.75% PPA is recommended based on a comprehensive analysis of the performance of compound modified asphalt, respectively, which can be equivalent to 6% SBS/SBR modified asphalt with high-temperature properties, low-temperature properties, temperature sensitivity and aging properties.

## 1. Introduction

Bituminous pavement has become the main highway pavement structure due to its characteristics of good smoothness, low noise, safe driving and ease of mechanized construction. With growing traffic volumes and various natural environments, a variety of pavement stresses such as ruts, cracks and water damage have emerged [1,2,3]. Many researchers came to the conclusion that it is needed modified asphalt binders to resist against distresses and improve the performance and longevity of bituminous pavement [4,5,6,7].

In recent years, the most commonly used asphalt modifiers are polymers such as styrene-butadiene-styrene (SBS), styrene-butadiene rubber (SBR), crumb rubber (CR) and polyethylene (PE). Among polymers, SBS polymer modification could be the most accepted and wide application all over the world. Many studies showed that the addition of SBS to the asphalt increases the softening point, decreases the penetration slightly, lessens the thermal susceptibility, increases the viscosity and decreases the Frass breaking point [8]. However, the SBS polymer is not chemically stable and vulnerable to aging due to a number of unsaturated double carbon bonds inside SBS molecular [9]. Meanwhile, SBR is a kind of synthetic rubber that can improve the elastic recovery and low-temperature ductility of asphalt binders. Furthermore, it could apply as a modifier along with other modifiers to produce compound modified binders [10]. According to Zhang and Yu, their results indicated that the addition of SBR could improve the thermal cracking resistance of the Polyphosphoric acid (PPA) modified binders [11]. However, the limited effect of SBR on asphalt high temperature performance makes it difficult to meet the demand for applying in hot areas. In addition, the great discrepancy in structure and solubility parameter between SBR and asphalt leads to potential instability for blends [12].

Polyphosphoric acid (PPA) is a polymer of orthophosphoric acid (H_3_PO_4_) which can be used alone and also with a combination of other additives in the modification of neat binders [4,13]. Past experience has shown PPA increases the high temperature stiffness of an asphalt binders with only minor effects on the intermediate and low temperature properties [14]. One of the first patents involved adding PPA to the asphalt binders to increase viscosity without increasing the penetration for asphalt modification was in 1973 [13]. Nunez et al. Their indicated that the addition of PPA into asphalt could increase its fatigue and rutting resistance [15]. Ho et al. studied the temperature sensitivity of PPA modified asphalt with different contents. The results showed that PPA has little effect on the low-temperature properties of asphalt [16]. Zhou Yan et al. evaluated the influence of PPA on the low-temperature properties of asphalt through 5 °C ductility test and beam bending creep test, and these results demonstrated that the modification effect of PPA on the low-temperature properties of asphalt was affected by the chemical composition of asphalt [17]. Ramasamy, B.S. et al. compared the rheological properties and aging properties of polymer modified asphalt and neat asphalt, their results showed that PPA can significantly improve the stiffness modulus and aging resistance of asphalt [18]. Based on the low price of PPA, the utilization of PPA to make SBR-modified binders of lower amount of SBR percentage. Peng Liang et al. comprehensively analyzed the influence of PPA on the rheological properties of SBR modified asphalt at high and low temperatures. The outcomes revealed that the gelation of PPA can improve the bonding between asphalts and enhance their elastic properties [12]. According to John D’Angelo, the multistress creep and recovery test were conducted to evaluate the interaction of PPA and polymer modification. The testing demonstrated that there is an interaction between PPA and SBS polymers which improved the high-temperature stiffness of the SBS-PPA compound modified asphalt [13]. Darrell Fee et al. studied the rheological properties of PPA compound SBS modified asphalt at low and high temperatures. The results showed that the performance was not obvious at low temperatures [19]. In a word, researchers have reached a relatively unified understanding of the high-temperature properties of PPA modified asphalt.

Considering the characteristics of PPA, SBS and SBR modified asphalt binders, the primary objective of this study is to investigate partially replacing SBS/SBR with PPA as asphalt modifiers, which can not only achieve lower cost, but also show better performances such as rutting resistance, high-temperature properties and anti-aging. To achieve the objective, the specific work is to investigate the effects of different PPA content on high-temperature properties, low-temperature properties, temperature sensitivity and aging properties of SBS/SBR modified asphalt binders using Dynamic Shear Rheometer (DSR), Bending Beam Rheometer (BBR) and Rolling Thin Film Oven (RTFO) testing.

## 2. Materials and Methods

### 2.1. Materials

#### 2.1.1. Asphalt

In order to analyze the effect of PPA on the properties of different neat asphalt, two types of neat asphalt widely used in China were selected in this study: DH-70# produced by SINOPEC (Beijing, China) and LH-90# produced by Liaohe Petrochemical Asphalt Corporation (Panjin, China) (the number represents the penetration grade). The asphalt technical indexes were tested according to the relevant test methods in the “*Standard Test Methods of Bitumen and Bituminous Mixtures for Highway Engineering*” (JTG E20-2011).The results are listed in Table 1.

#### 2.1.2. PPA

The 110% PPA (PPA-110) was produced by Nanjing Chemical Reagent Corporation (Nanjing, China) and was selected for the preparation of modified asphalt.

#### 2.1.3. Polymer Modifier

Two types of polymer modifiers were selected: SBS1401(YH-792, linear) produced by SINOPEC (Beijing, China) and SBR produced by Beijing Yudahang Industry and Trade Corporation (Beijng, China). The basic technical indexes are listed in Table 2.

#### 2.1.4. Schemes of Modified Asphalt with PPA and SBS/SBR Compound Ratio

The SBS/SBR content (by weight of neat asphalt) of SBS/SBR modified asphalt is usually 3–6%, and the PPA content of polymer modified asphalt is generally 0.5–1.5% [20,21]. Different contents of SBS/SBR and PPA were used to prepare different modified asphalt binders. Based on the recommendations of the past studies, the polymer content was set as 4%, and the PPA content was 0.5%, 0.75%, 1.0%, 1.25% and 1.5% respectively. Seven test schemes are shown in Table 3.

### 2.2. Specimen Preparation

The following process is adopted for the preparation of PPA and SBS/SBR compound modified asphalt:

(1) The neat asphalt was heated to 150 °C until it became fluid before mixing;
(2) The weighed SBS/SBR particles were added into the asphalt and conducted high-speed shear at the speed of 4500 rpm for 30 min;
(3) Then, the weighed PPA was added into the prepared modified asphalt and conducted high-speed shear at the speed of 4500 rpm for 30 min;
(4) The sheared PPA and SBS/SBR compound modified asphalt were put into an oven at 180 °C for swelling and developing for 1 h; therefore, PPA and SBS/SBR compound modified asphalt was prepared, then the prepared PPA and SBS/SBR compound modified asphalt was poured into corresponding containers for storage.

### 2.3. Experimental Program

#### 2.3.1. DSR Test

The Physica MCR 301 DSR was used to investigate the high-temperature properties of the compound modified asphalt binders. The test modes include temperature sweep test and frequency sweep test to obtain the phase angle (δ) reflecting the viscoelasticity of asphalt and the complex shear modulus (G*) reflecting the high temperature stability of asphalt [22]. The loading frequency of temperature sweep test was 1.59 Hz [23]. Considering that in the frequency sweep test, the driving speeds of 8~16 and 80~100 km/h correspond to the frequencies of 0.15 and 1.5 Hz, respectively, in order to cover the driving speed range of vehicles specified on roads, the frequency are set at 0.01~16.0 Hz [24]. The test temperature of frequency sweep test is 60 °C. The viscosity temperature index (VTS) method and complex modulus index (GTS) method can be used to evaluate asphalt temperature sensitivity based on DSR test. In this study, G*, δ and other rheological parameters of each sample was tested to evaluate the temperature sensing performance of modified asphalt binders.

#### 2.3.2. BBR Test

The TE-BBR was used to evaluate the low-temperature performance of asphalt binders. Small beam specimens were prepared and tested to obtain the creep rate m-value and creep stiffness (S) under constant load at different temperatures. The m-value and S were used as the evaluation indexes of asphalt low-temperature performance. The asphalt binder with low S value and high m-value showed better low-temperature properties [25].

#### 2.3.3. RTFO Test

The Rolling thin film oven (RTFO) test was used to simulate the short-term aging of asphalt. The heating temperature was kept at 163 ± 0.5 °C, and the penetration, softening point and asphalt quality of PPA modified asphalt with different contents are tested before and after aging.

## 3. Results and Discussion

### 3.1. High-Temperature Properties

#### 3.1.1. Temperature Sweep Test

SHRP proposes to adopt rutting factor G*/sin δ to characterize the ability of asphalt to resist high temperature rutting deformation, the larger G*/sin δ, the stronger the rutting resistance of asphalt. G* is the complex shear modulus, which is the ratio of the maximum shear stress to the maximum shear strain of the material, and represents the measurement parameter of the total deformation resistance of the material under repeated shear load; δ is the phase angle, which represents the viscoelastic characteristics of the material, most materials are between ideal elasticity and ideal viscosity. The smaller δ, the stronger the elasticity of the material.

**Figure 1:**
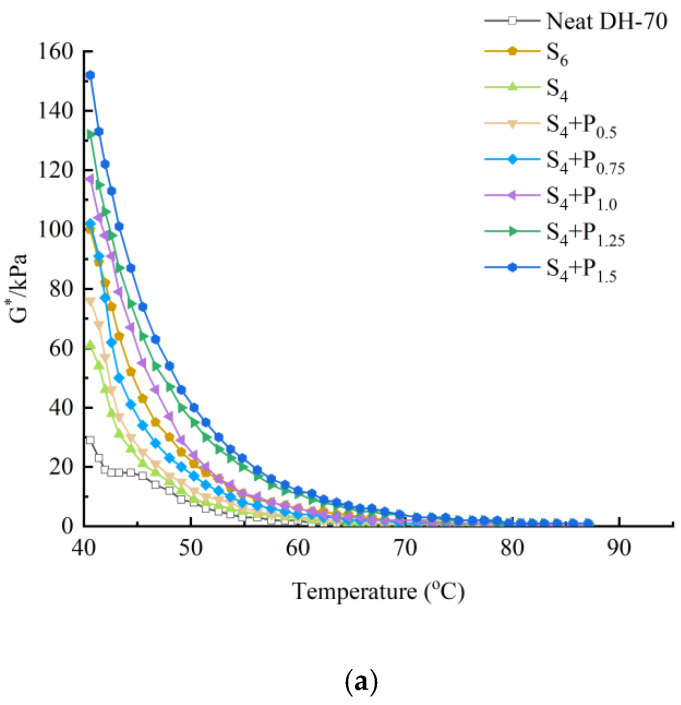

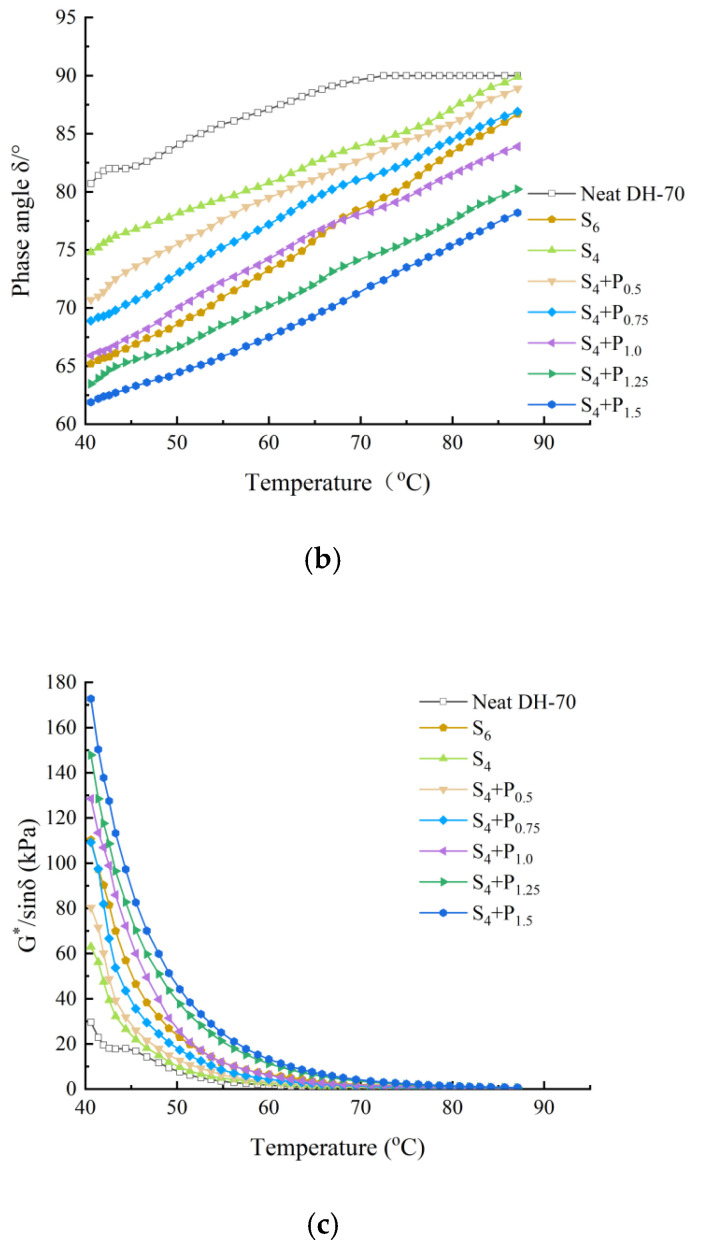
High-temperature properties of compound ratio of PPA–SBS and DH–70# neat asphalt: (**a**) Complex modulus-temperature results; (**b**) Phase angle-temperature results; (**c**) G*/sinδ-tenperature results.

**Figure 2:**
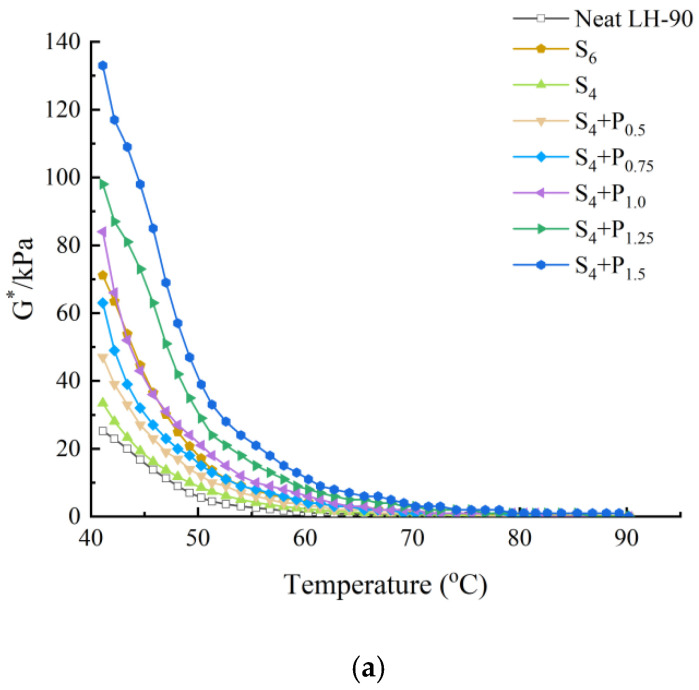

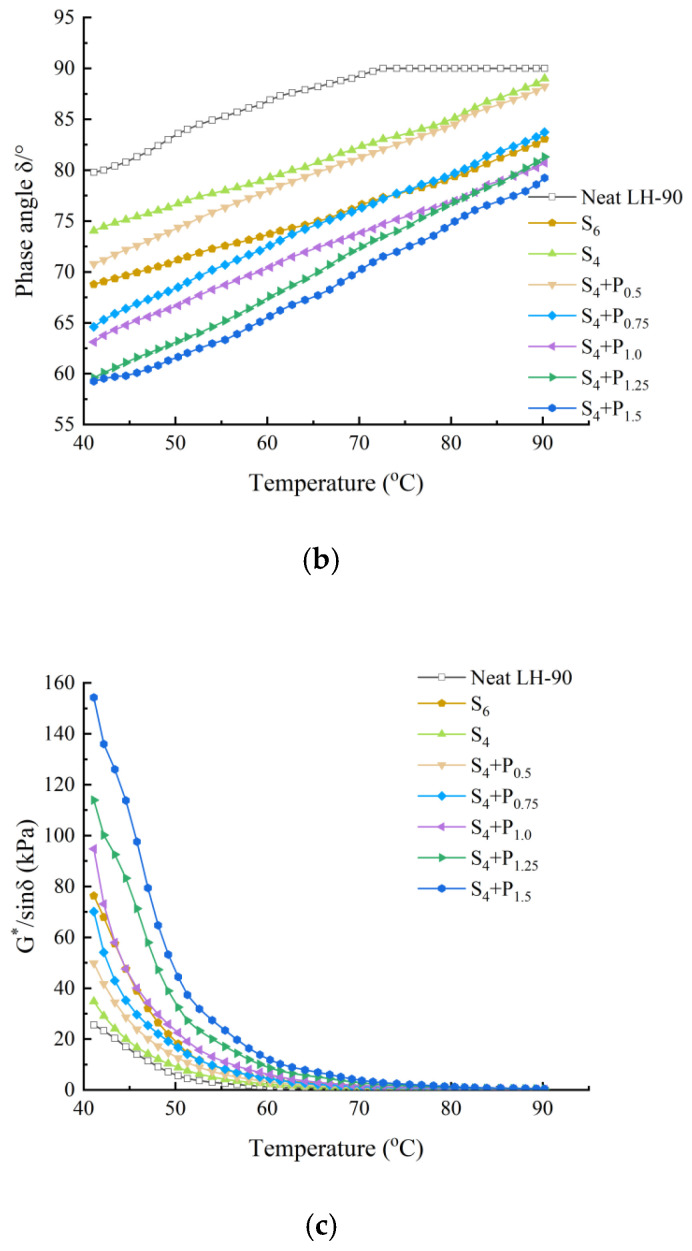
High-temperature properties of compound ratio of PPA–SBS and LH–90# neat asphalt: (**a**) Complex modulus-temperature results; (**b**) Phase angle-temperature results; (**c**) G*/sinδ-tenperature results.

**Figure 3:**
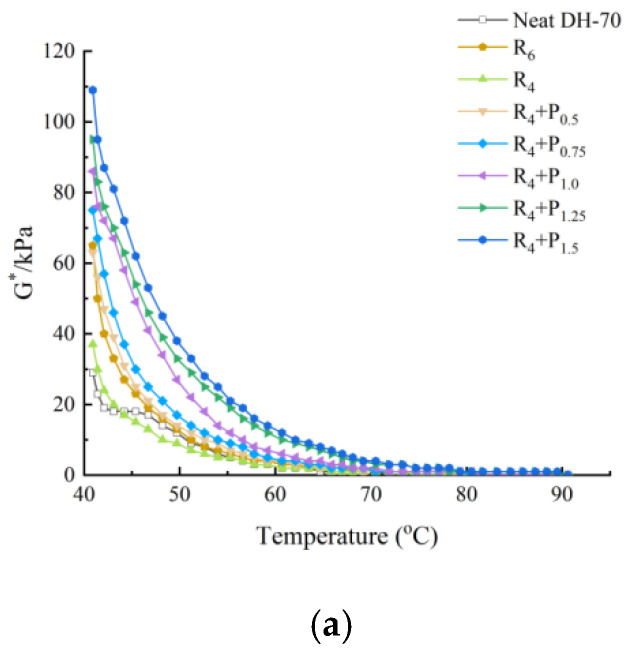

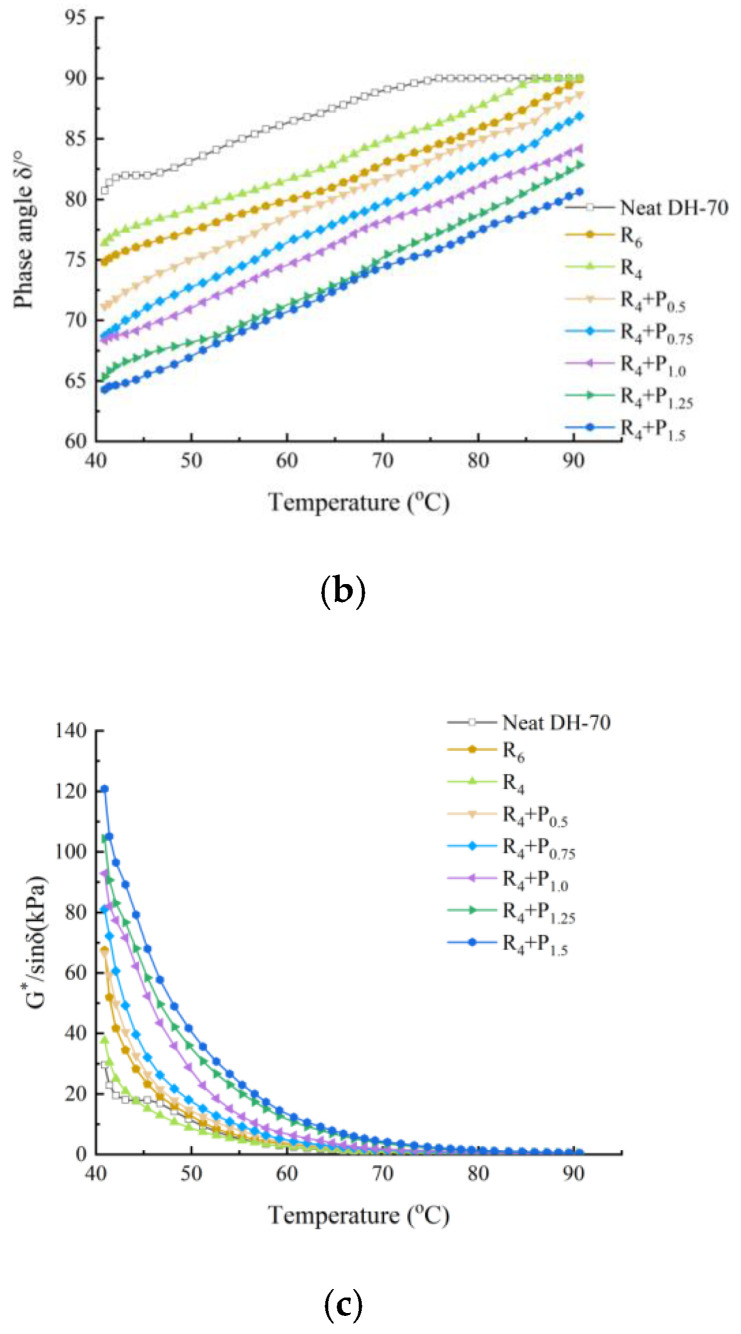
High-temperature properties of compound ratio of PPA–SBR and DH–70# neat asphalt: (**a**) Complex modulus-temperature results; (**b**) Phase angle-temperature results; (**c**) G*/sinδ-tenperature results.

**Figure 4:**
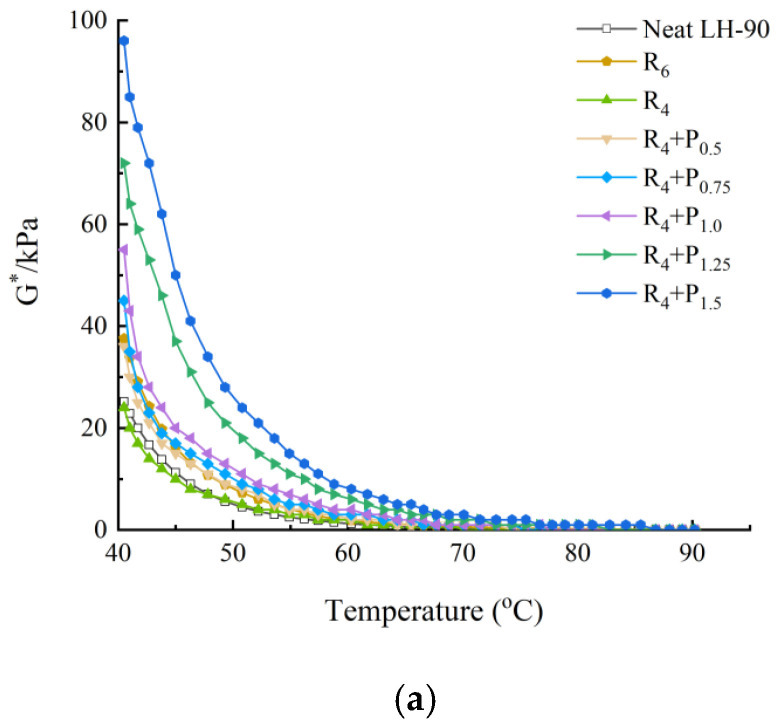

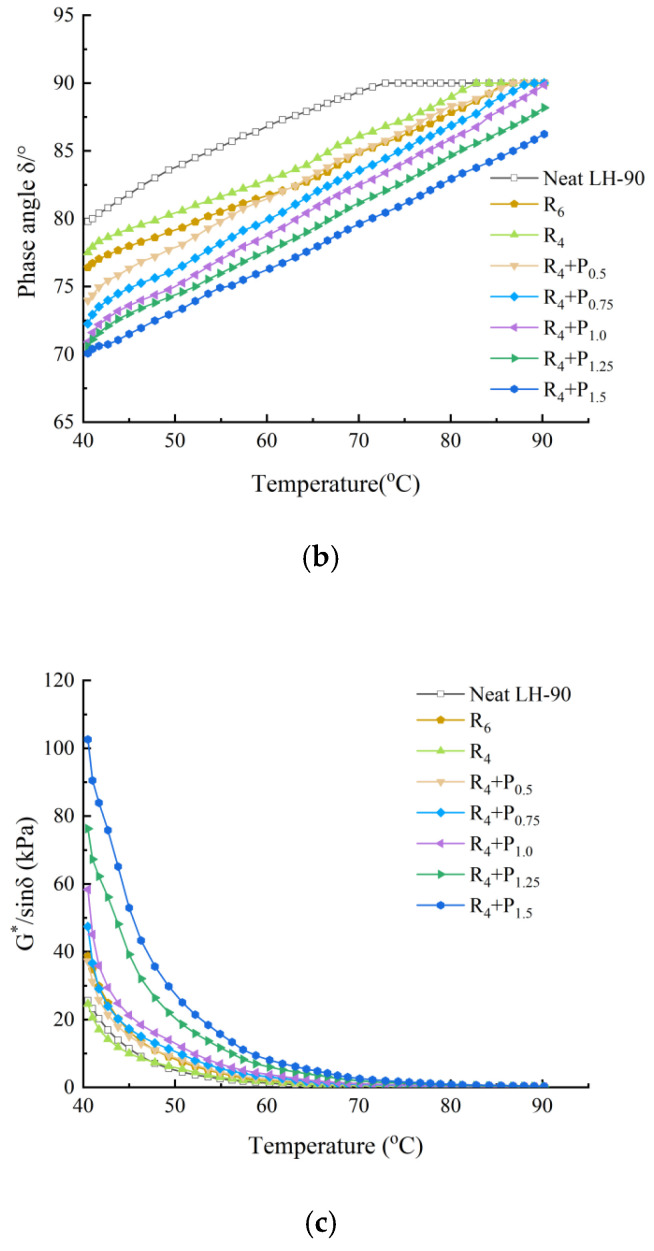
High-temperature properties of compound ratio of PPA–SBR and LH–90# neat asphalt: (**a**) Complex modulus-temperature results; (**b**) Phase angle-temperature results; (**c**) G*/sinδ-tenperature results.

This can be seen in Figure 1, Figure 2, Figure 3 and Figure 4.

Under the same neat asphalt type and temperature, adding PPA modifier can significantly improve the G* of modified asphalt, reduce the phase angle δ, it shows that PPA can reduce the viscosity characteristics at the same temperature, improve the elastic characteristics of modified asphalt and enhance the shear deformation resistance of asphalt at high temperature. The larger the content of PPA, the more obvious the modification effect is.

The rutting factor G*/sinδ decreases with the increase of temperature.S_6_ shows higher G*/sinδ value than S_4_ and R_6_ shows higher G*/sinδ value than R_4_. At the same temperature, by comparing the G*/sinδ values of SBS-PPA and SBR-PPA compound modified asphalt binders, it can be found that the G*/sinδ increased with the increase of PPA concentration, indicating that PPA can improve the rutting resistance ability of the SBS/SBR modified asphalt binders

The same high-temperature properties can be achieved through replacing 2% SBS with 0.75–1.0% PPA or replacing 2% SBR with 0.5% PPA, which also cost less.

#### 3.1.2. Frequency Sweep Test

Asphalt will show different viscoelastic characteristics and mechanical states when the temperature and load frequency change. Asphalt with more stiff and elastic characteristics under high temperature conditions can have better rutting resistance.

**Figure 5:**
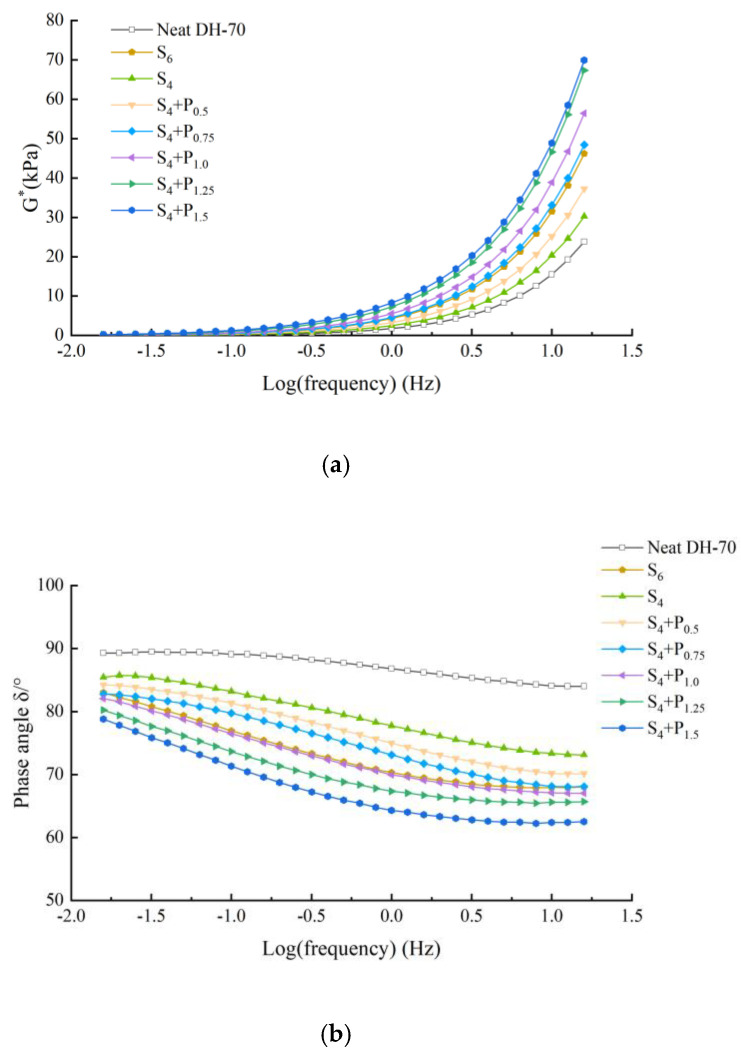
Complex modulus and phase angle of compound PPA–SBS modified binders of DH–70# asphalt: (**a**) Complex modulus-Log(frequency) results; (**b**) Phase angle-Log(frequency) results.

**Figure 6:**
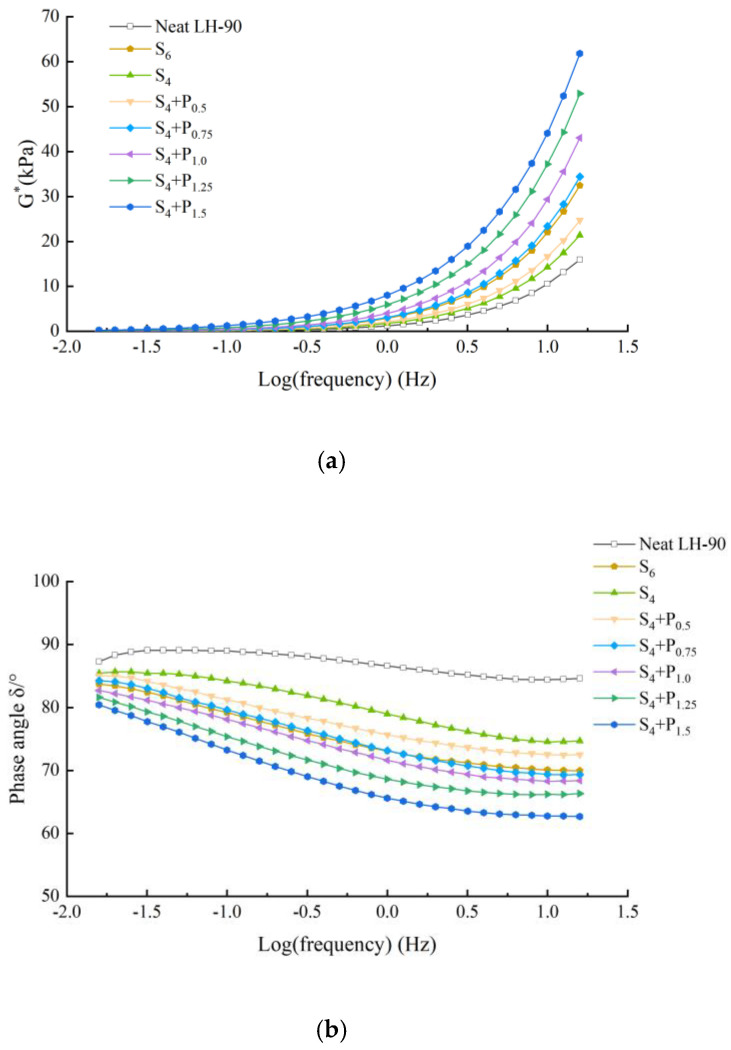
Complex modulus and phase angle master of compound PPA–SBS modified binders of LH–90# asphalt: (**a**) Complex modulus-Log(frequency) results; (**b**) Phase angle-Log(frequency) results.

**Figure 7:**
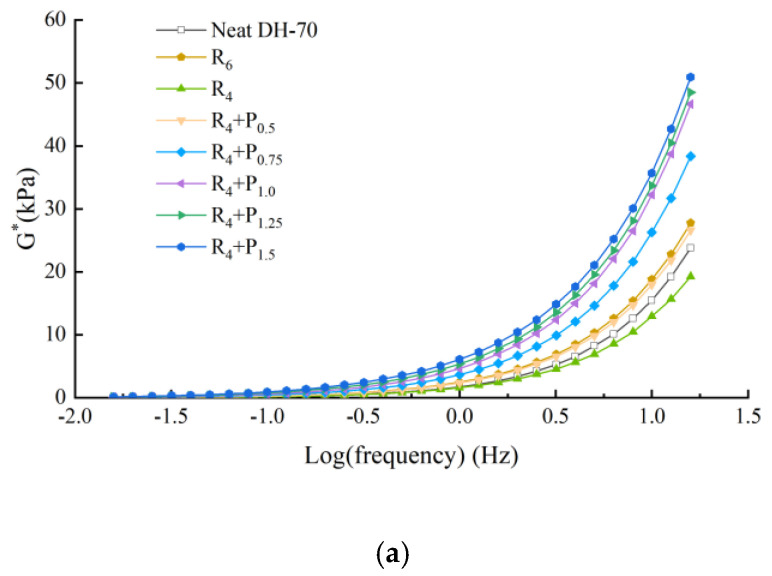

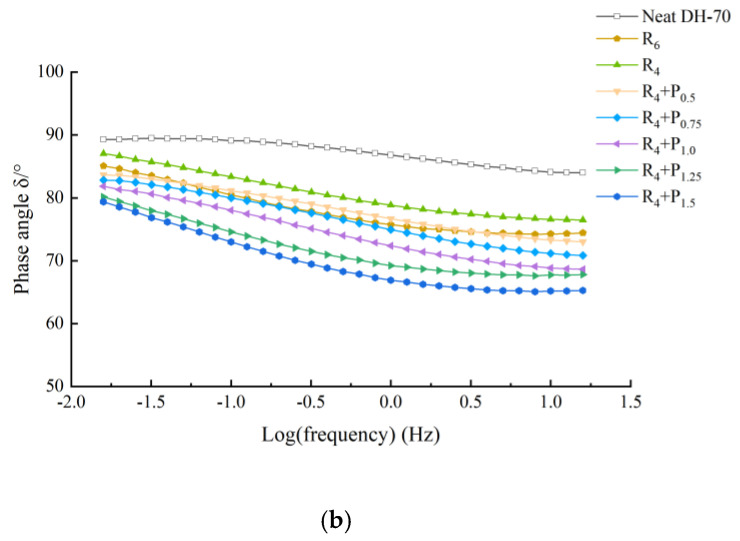
Complex modulus and phase angle master of compound PPA–SBR modified binders of DH–70# asphalt: (**a**) Complex modulus-Log(frequency) results; (**b**) Phase angle-Log(frequency) results.

**Figure 8:**
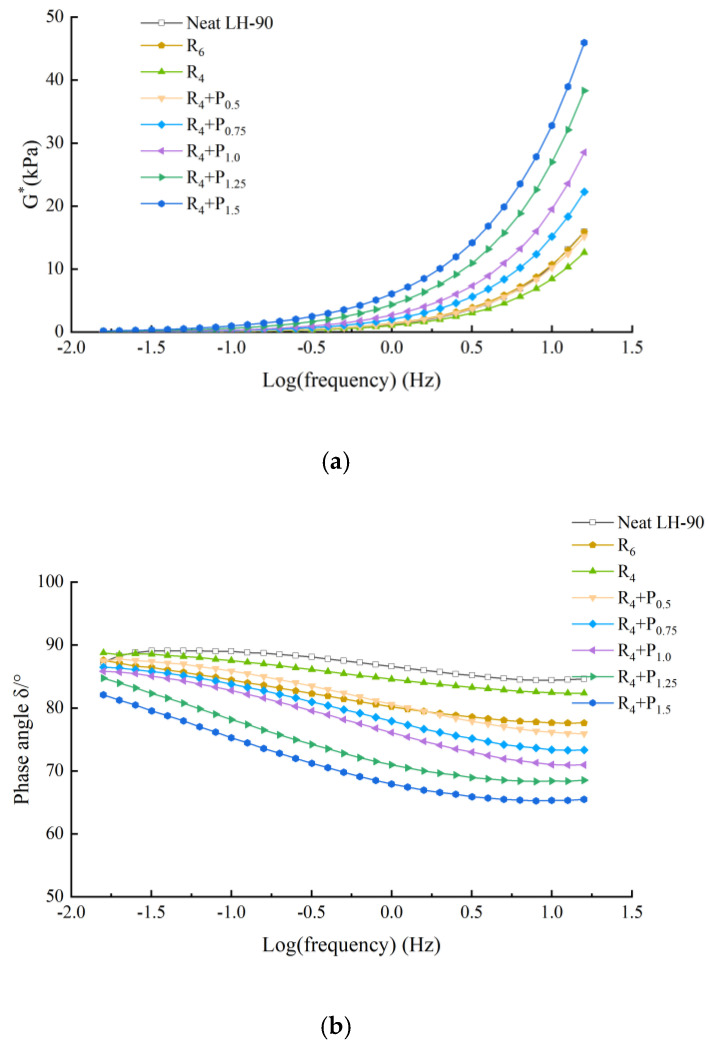
Complex modulus and phase angle master of compound PPA–SBR modified binders of LH–90# asphalt: (**a**) Complex modulus-Log(frequency) results; (**b**) Phase angle-Log(frequency) results.

The frequency sweep test was performed to identify the mechanical and viscoelastic behavior of asphalt binder. Figure 5, Figure 6, Figure 7 and Figure 8 illustrate the complex modulus and phase angle of compound modified binders with different SBS/SBR and PPA concentrations. The G* studied by Superpave can be decomposed into two components: storage modulus G’ and loss modulus G’’. The G’ represents the elastic part of asphalt binder, and the G’’ represents the viscous part of asphalt binder [26]. It reflects the energy loss part of asphalt during deformation. At high temperature, the greater the G’, the stronger the high temperature stability of the binder is. At low temperature, the greater the G’’, the stronger the crack resistance of the binder is [27].

This can be seen in Figure 5, Figure 6, Figure 7 and Figure 8.

Complex modulus values increasing and phase angle values decreasing can be observed for all the tested asphalt binders. The curves of the two binders show increasing complex modulus and decreasing phase angle for higher content of PPA addition, which indicates transition of asphalt binder from a viscoelastic to elastic state.

At a specific frequency, by comparing the complex modulus and phase angle of SBS-PPA or SBR-PPA compound modified asphalt binders with that of 6% SBS/SBR single modified asphalt, the compound modified asphalt schemes with similar performance to these single modified asphalts can be obtained, which are listed in Table 4.

Compared with the base asphalt, the addition of modifier effectively improves the elastic characteristics and the total deformation resistance of asphalt under the same load frequency. Considering the price of PPA, it is more recommended to replace 2% SBS/SBR with 0.75/0.5% PPA for DH-70# asphalt, and replace 2% SBS/SBR with 0.5% PPA for LH-90# asphalt.

### 3.2. Low-Temperature Properties

**Figure 9:**
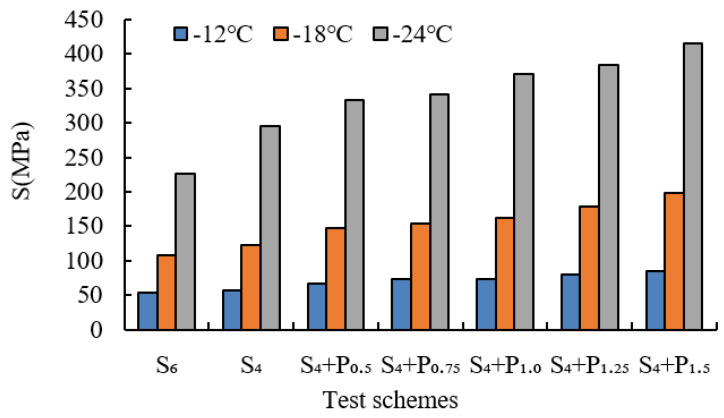
Creep stiffness of PPA–SBS and DH–70# asphalt.

**Figure 10:**
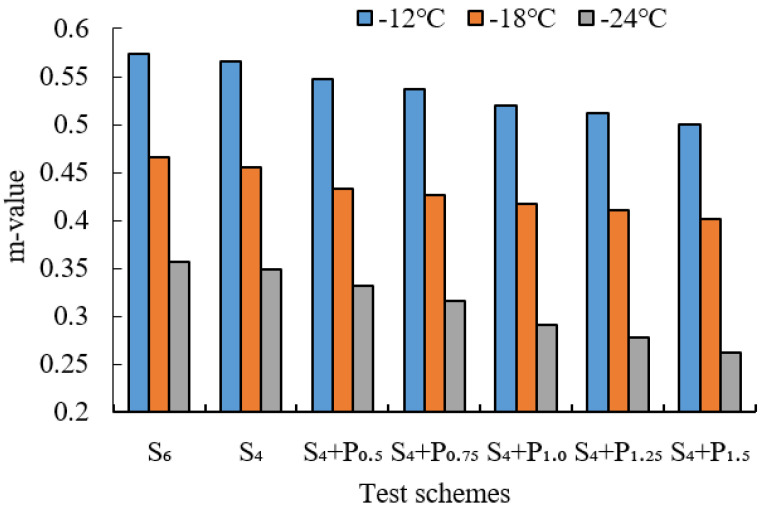
m-value of PPA–SBS and DH–70# asphalt.

**Figure 11:**
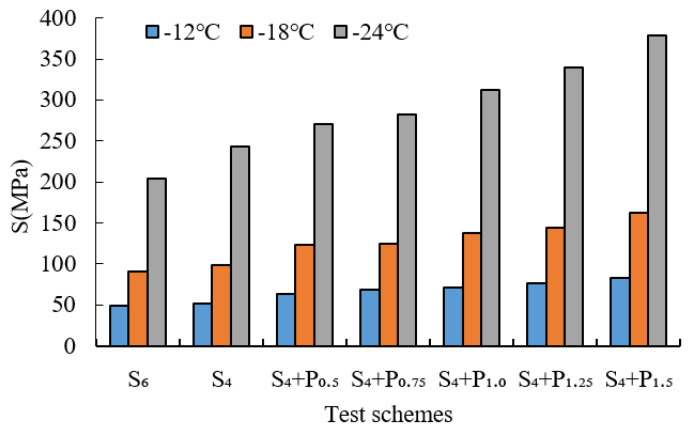
Creep stiffness of PPA–SBS and LH–90# asphalt.

**Figure 12:**
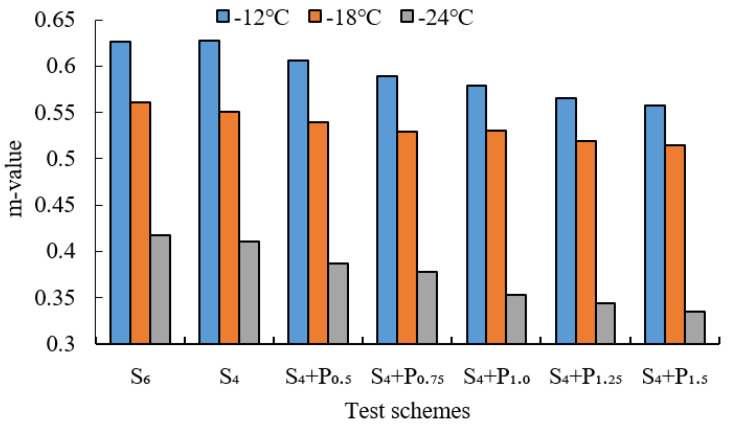
m-value of PPA–SBS and LH–90# asphalt.

**Figure 13:**
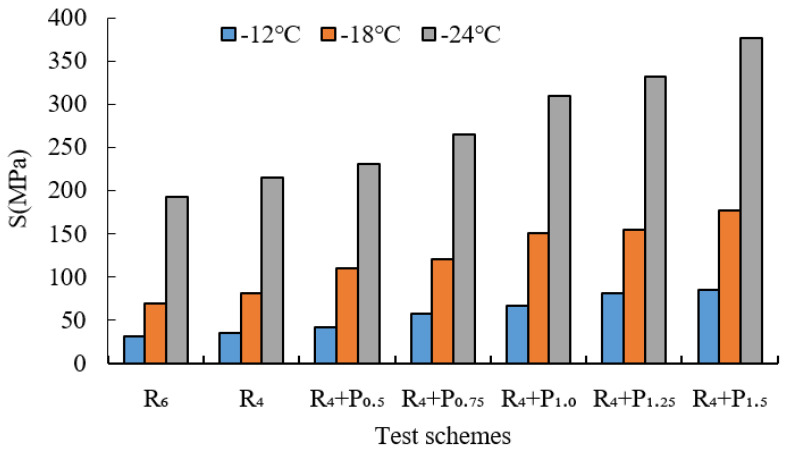
Creep stiffness of PPA–SBR and DH–70# asphalt.

**Figure 14:**
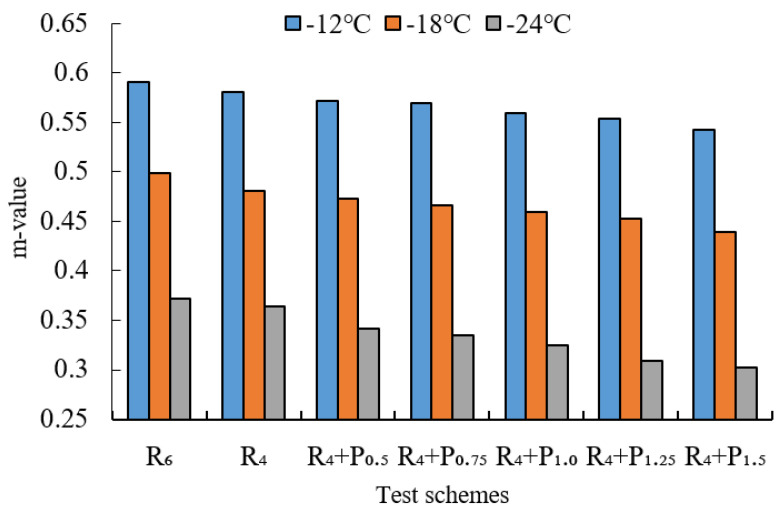
m-value of PPA–SBR and DH–70# asphalt.

**Figure 15:**
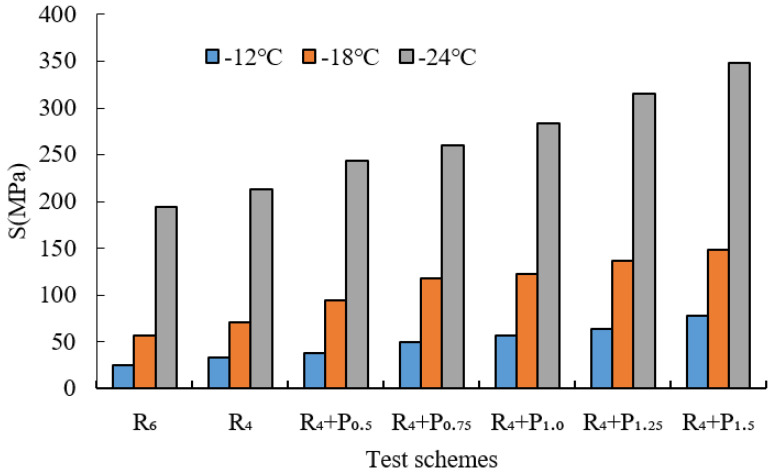
Creep stiffness of PPA–SBR and LH–90# asphalt.

**Figure 16:**
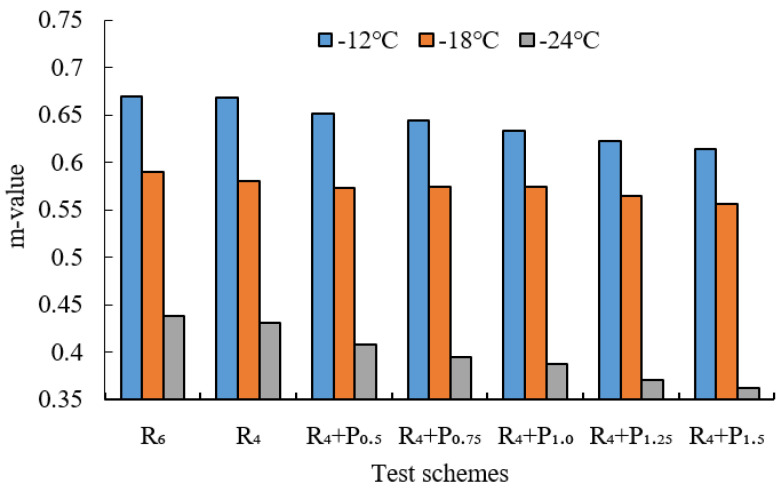
m-value of PPA–SBR and LH–90# asphalt.

BBR tests were conducted to evaluate the low-temperature cracking resistance of the studied modified asphalt binders. The tests were carried out at different temperatures (−12 °C, −18 °C and −24 °C) and the creep stiffness(S) and m-value were evaluated. Creep stiffness(S) represents the resistance load performance of asphalt. The creep rate m-value is the absolute value of the slope of the logarithm of the stiffness curve versus the logarithm of time and reflects the stress relaxation ability of asphalt at low temperature. The test results are shown in Figure 9, Figure 10, Figure 11, Figure 12, Figure 13, Figure 14, Figure 15 and Figure 16.

For grading of the low-temperature performance of asphalt binders, the Superpave limits that the specified maximum creep stiffness value at 60 s is 300 MPa, i.e., S < 300 MPa, and the creep rate to a minimum of 0.30, i.e., m value > 0.3, at 60 s. As shown in Figure 9, Figure 10, Figure 11, Figure 12, Figure 13, Figure 14, Figure 15 and Figure 16, The S of 6% SBS is lower (or similar) than 4% SBS, the m-value of 6% SBS is higher (or similar) than 4% SBS, and SBR has the same results. However, with the addition of PPA, the S gradually increases and the m-value gradually decreases. The results indicate that PPA has a negative impact on the low-temperature properties and stress relaxation ability of SBS/SBR modified asphalt, which significantly reduces the low-temperature ductility of SBS/SBR modified asphalt.

For SBR-PPA compound modified asphalt binders of LH-90# asphalt at −24 °C, the m-value is higher than 0.3, which indicates that the low-temperature performance grade decreased one level. The S of some asphalts are higher than 300 MPa at −24 °C with the addition of PPA. In order to guarantee the low-temperature properties of asphalt, PPA content should be no more than 1.0%, and service temperature cannot less than −18 °C.

### 3.3. Temperature Sensitivity

#### 3.3.1. Complex Modulus Index (GTS) Method

SHRP proposes to use complex modulus index (GTS) to test the temperature sensitivity of asphalt under medium temperature to high temperature conditions [28]. GTS is calculated by fitting the double logarithm of complex modulus G* with the logarithm of test temperature T. Equation (1) presents the definition of GTS.

(1) $$\mathrm{lg}[\mathrm{lg}(G^{*})]\;=\;\mathrm{GTS}\;\times \;\mathrm{lg}(T)\;+\;C$$

where G* is the complex shear modulus, T is the test temperature, C is the constant term in regression fitting, GTS is the complex modulus index.

**Figure 17:**
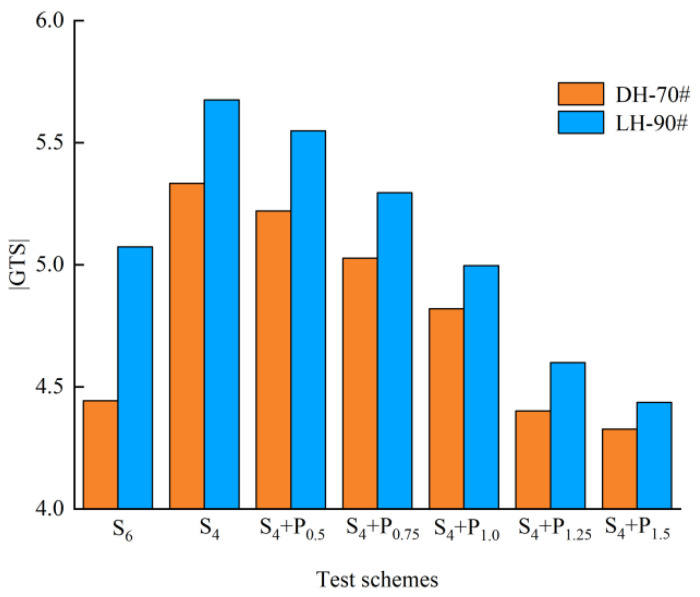
Effect of different PPA–SBS compound ratio on temperature sensitivity of asphalt.

**Figure 18:**
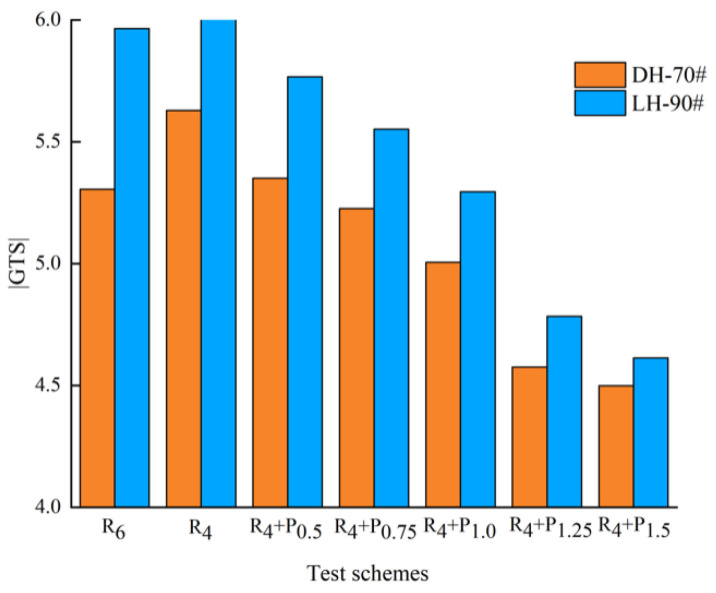
Effect of different PPA–SBR compound ratio on temperature sensitivity of asphalt.

Considering that the pavement surface temperature is usually up to 60 °C or higher in summer, temperature sweep tests were conducted at 58 °C, 64 °C, 70 °C and 76 °C, and the GTS results are shown in Figure 17 and Figure 18.

As shown in Figure 17 and Figure 18, the |GTS| of S_6_ is lower than S_4_, and SBR has the same results. With the addition of PPA, the |GTS| of modified asphalt gradually decreases. The results indicate that the temperature sensitivity of compound modified asphalt is improved. For DH-70 # asphalt, the | GTS | values of S_4_ + P_1.25_ shows similar to S_6_ and R_4_ + P_0.5_ shows similar to R_6_. For LH-90# asphalt, the |GTS| values of S_4_ + P_1.0_ shows similar to S_6_ and R_4_ +P_0.5_ shows similar to R_6_.

In order to decrease costs and reduce the temperature sensitivity of asphalt binder, replacing 2% SBS/SBR with 1.25%/0.5% PPA for DH-70# asphalt, and replacing 2% SBS/SBR with 1.0%/0.5% PPA for LH-90# asphalt is more recommended, respectively.

#### 3.3.2. Viscosity Temperature Index (VTS) Method

The results of many domestic scholars show that [29], it is more appropriate to use Kelvin temperature scale to calculate viscosity temperature index VTS to evaluate the temperature sensitivity of modified asphalt. Low temperature sensitivity of asphalt binder indicates better asphalt pavement performance. The viscosity of asphalt could be obtained by Equation (2), it refers to the ability of asphalt material to resist shear deformation under the action of external force. and VTS could be obtained by Equation (3). Curve fitting is performed to show the correlation between viscosity and temperature and, from which, the slopes (VTS) and coefficient of determinations (R^2^) are obtained.

(2) $$\eta =\frac{G^{*}}{\omega }{\left(\frac{1}{\sin\delta }\right)}^{4.8628}$$

(3) $$\mathrm{VTS}=\frac{\mathrm{lg}(\mathrm{lg}\eta_{1})-\mathrm{lg}(\mathrm{lg}\eta_{2})}{\mathrm{lg}T_{1}-\mathrm{lg}T_{2}}$$

where η is the viscosity, ω is the loading frequency, ω = 10 Hz, η1 and η2 is the viscosity corresponding to the adjacent temperature, T is Kelvin temperature, T = t + 273.13, t is Celsius temperature, which is 58 °C, 64 °C, 70 °C and 76 °C.

**Figure 19:**
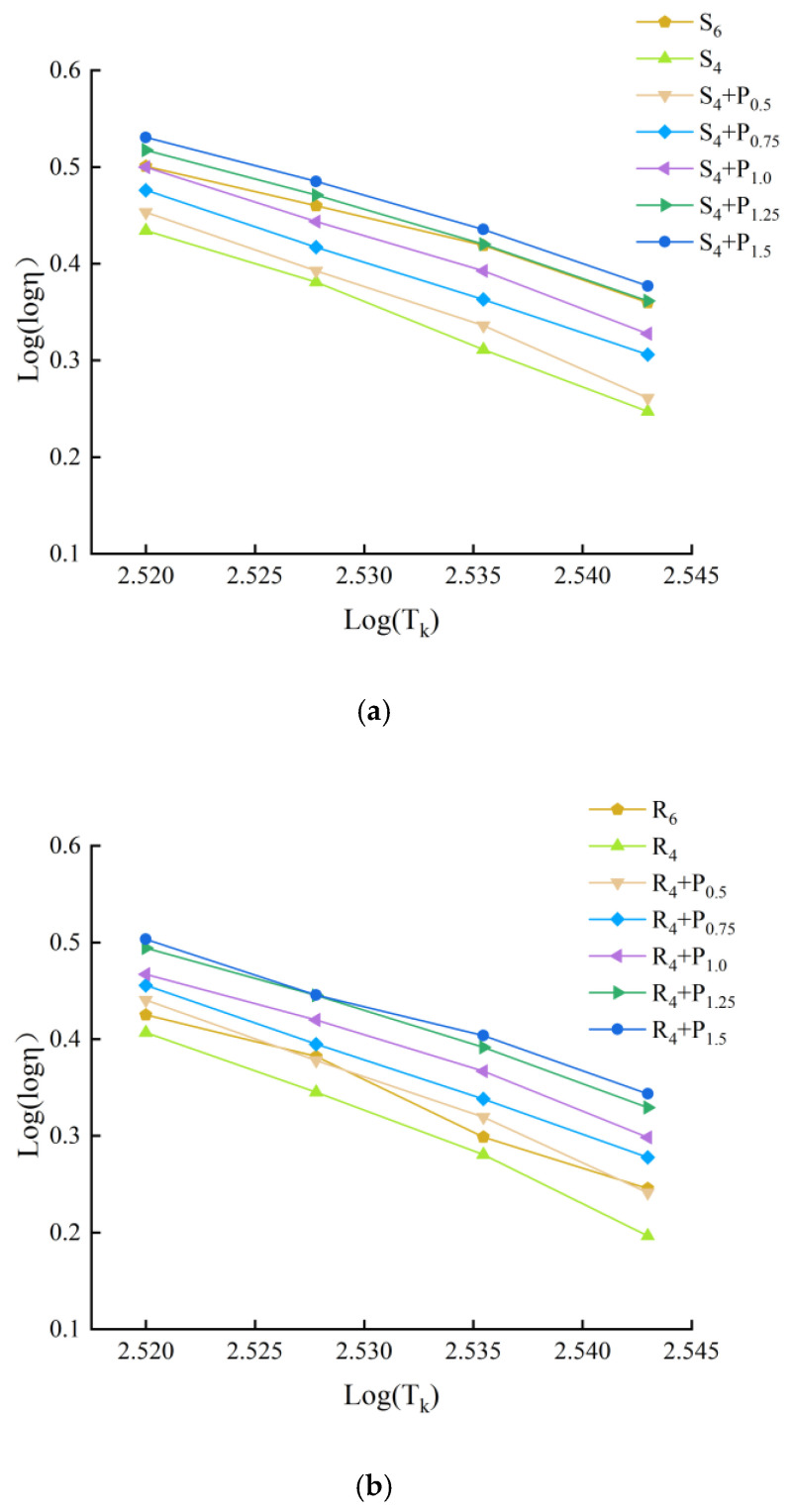
Double logarithm regression viscosity temperature curve of DH–70# asphalt: (**a**) PPA–SBS compound ratio; (**b**) PPA–SBR compound ratio.

**Figure 20:**
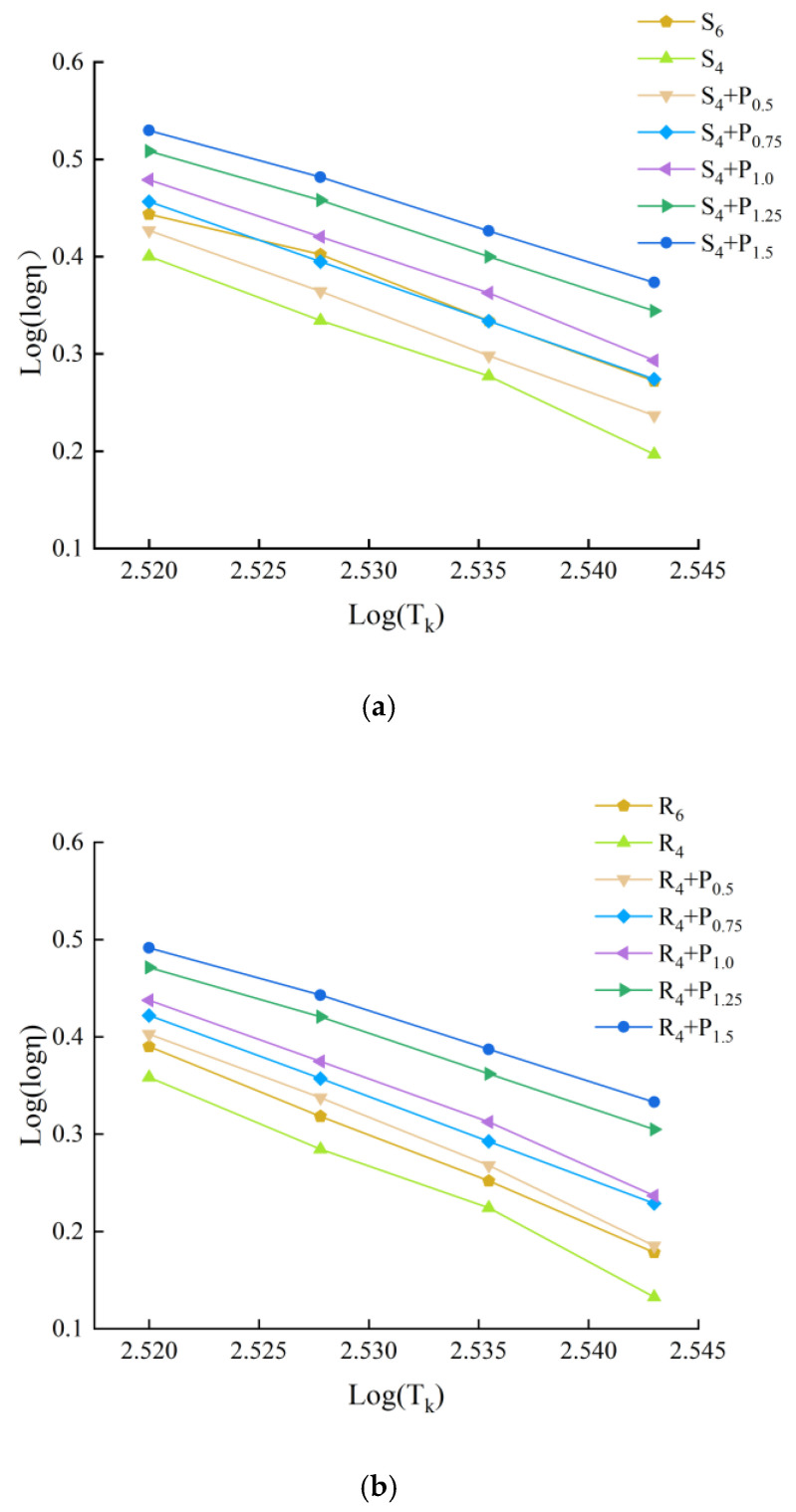
Double logarithm regression viscosity temperature curve of LH–90# asphalt: (**a**) PPA–SBS compound ratio; (**b**) PPA–SBR compound ratio.

**Figure 21:**
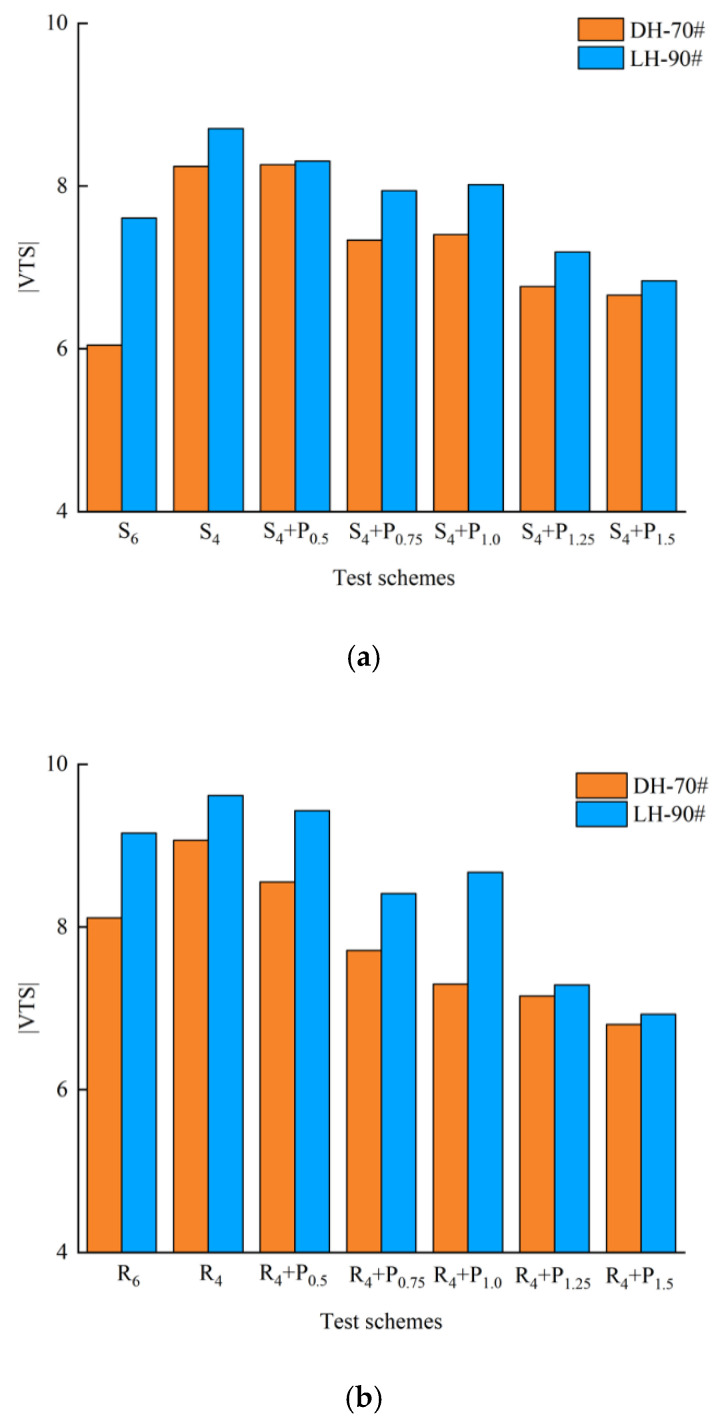
SBS/SBR-PPA variation curve of |VTS|: (**a**)|VTS| of PPA–SBS with DH–70# and LH–90#; (**b**)|VTS| of PPA–SBR with DH–70# and LH–90#.

According to Equation (2), logarithmic regression is carried out for different compound asphalt at different temperatures and corresponding viscosity. The slope of the regression equation is the viscosity temperature change rate. The greater its absolute value, the worse the temperature sensitivity of asphalt. The regression curve and regression results are shown in Figure 19, Figure 20 and Figure 21 and Table 5.

Table 5 illustrates the slopes |VTS| and the coefficient of determinations (R^2^), respectively. The R^2^ value is greater than 0.99, which indicates that lg(lg(viscosity)) has a good linear correlation with lg(T). The larger the magnitude of the VTS value is found to be, the more susceptible the binder is to changes in viscosity with temperature [30]. It can be seen that the slope value of modified asphalt binders integrally decreases with the more PPA concentration, indicating that PPA contributes to the reduction in temperature sensitivity.

To obtain similar sensitive properties, replacing 2% SBS/SBR with 1.5%/0.5% PPA for DH-70# asphalt, and replacing 2% SBS/SBR with 0.75%/0.5% PPA for LH-90# asphalt is more recommended, respectively.

### 3.4. Aging Properties

In this study, RTFO test was used to simulate the short-term aging effect and test the performance changes of PPA compound modified asphalt before and after aging. The heating temperature was 163 ± 0.5 °C. The Retained penetration ratio, Softening point increment and Mass loss rate results are shown in Table 6 and Table 7.

As shown in Table 6 and Table 7, the compound modified binders with S_6_ shows higher retained penetration ratio (RP) value than those with S_4_, and the binder with higher SBS concentration shows lower value softening point increment (∆S) and mass loss rate (∆M) than that with lower concentration. Furthermore, SBR has the same results.PPA shows a significant effect on RP, ∆S and ∆M. With RTFO aging, RP value increases with the increase of PPA, but ∆S and ∆M decrease. This indicates that PPA is less prone to oxidation to improve the anti-aging properties of SBS/SBR modified asphalt.

In addition, for DH-70 # asphalt, the aging indices of S_4_ + P_1.0_ and S_6_ show similar values and the aging indices of R_4_ + P_0.75_ and R_6_ show similar values. For LH-90 # asphalt, the aging indices of S_4_ + P_0.75_ and S_6_ show similar values and the aging indices of R_4_ + P_0.75_ and R_6_ show similar values.

Based on the above statements, to obtain the similar anti-aging properties, replacing 2% SBS/SBR with 1.0%/0.75% PPA for DH-70# asphalt, and replacing 2% SBS/SBR with 0.75% PPA for LH-90# asphalt is more recommended, respectively.

## 4. Conclusions

To investigate the feasibility of using PPA to partially replace SBS/SBR as asphalt modifier, through a series of tests, including DSR, BBR and RTFO tests, the performance of compound asphalt is improved compared with that of 4% SBS/SBR single modified asphalt. The results show that appropriate PPA content can improve the elastic performance of SBS/SBR modified asphalt, reduce its viscosity, and improve the high-temperature deformation resistance, therefore, the rutting resistance of compound asphalt is improved. After adding PPA, |GTS| decreases significantly and |VTS| decreases gradually, indicating that PPA reduces the temperature sensitivity of modified asphalt.

In addition, PPA can inhibit the thermal oxidative aging of polymer modified asphalt, thereby enhancing the anti-aging properties, and the greater the amount of PPA, the more significant the modification effect is.

Overall, the high temperature, temperature sensitivity and anti-aging properties of polymer modified asphalt were improved after adding PPA, and because of its low price, the polymer modified asphalt can reduce some polymer content by adding PPA. PPA may have an adverse effect on the stiffness and stress relaxation of asphalt binder, but it can meet the specification requirements in the area where the average temperature in winter is not lower than −18 °C. Considering those performance and economic benefits, the recommended scheme of PPA-SBS compound modified asphalt is 4% SBS + 0.75–1.0% PPA, and the recommended scheme of PPA-SBR compound modified asphalt is 4% SBR + 0.5–0.75% PPA, which is equivalent to the performance of 6% SBS/SBR single modified asphalt. Following research work should include investigating the combinations of PPA with other modifiers and neat asphalt, as well as the verification of field correlation, so as to prepare asphalt with better performance and wider application range.

## Figures and Tables

**Table 1:** Technical performance of asphalt.

	Technical Indexes	Units	Results	
			DH-70#	LH-90#
Unaged	Softening point(R&B)	°C	53.1	49.2
	Ductility (50 mm/min, 10 °C)	cm	39.7	>100
	Penetration (25 °C, 5 s, 100 g)	0.1 mm	64.2	84.6
	Solubility	%	99.9	99.7
	Density(15 °C)	g/cm^3^	1.033	1.042
	Flash point	°C	279	316
RTFO aged	Mass variation	%	0.47	−0.16
	Retained penetration ratio (25 °C)	%	70.2	73.1
	Retained ductility (10 °C)	cm	14.1	8.7

**Table 2:** Basic technical indexes of SBS and SBR.

SBS1401			SBR		
Technical Index	Units	Measured Value	Technical Index	Units	Measured Value
S/B ratio	-	4/6	Granularity	Mesh No.	10~80
Oil filling rate	%	0	Bound styrene content	%	10~50
Volatile matter	%	≤0.7	Mooney viscosity	ML	45~65
Ash content	%	≤0.2	300% Constant tensile stress	MPa	15
300% Constant tensile stress	Mpa	≥3.5	Tensile strength	Mpa	≥20
Tensile strength	Mpa	24			
Elongation at break	%	730			
Shore hardness	A	85			
Melt flow rate	g/10 min	0.1~5.0			

**Table 3:** Compound modification schemes of PPA.

No	Schemes
S_6_/R_6_	6% SBS/SBR Modification
S_4_/R_4_	4% SBS/SBR Modification
S_4_/R_4_ + P_0.5_	4% SBS/SBR + 0.5% PPA Modification
S_4_/R_4_ + P_0.75_	4% SBS/SBR + 0.75% PPA Modification
S_4_/R_4_ + P_1.0_	4% SBS/SBR + 1.0% PPA Modification
S_4_/R_4_ + P_1.25_	4% SBS/SBR + 1.25% PPA Modification
S_4_/R_4_ + P_1.5_	4% SBS/SBR + 1.5% PPA Modification

**Table 4:** Schemes of compound modified asphalt with similar performance to S6/R6.

Asphalt Type	Polymer Modifier Type	Complex Modulus G*/kPa	Phase Angleδ/°	Recommended Scheme
DH-70#	SBS	S_4_ + P_0.75_	S_4_ + P_1.0_	S_4_ + P_0.75_
	SBR	R_4_ + P_0.5_	R_4_ + P_0.5~0.75_	R_4_ + P_0.5_
LH-90#	SBS	S_4_ + P_0.75_	S_4_ + P_0.75_	S_4_ + P_0.75_
	SBR	R_4_ + P_0.5_	R_4_ + P_0.5~0.75_	R_4_ + P_0.5_

**Table 5:** Double logarithm regression results of different asphalt mixtures.

Asphalt Type	No	Regression Equation	Correlation Coefficient R^2^	|VTS|
DH-70#	S_6_	y = −6.0467x + 15.743	R^2^ = 0.9895	6.0467
	S_4_	y = −8.2404x + 21.204	R^2^ = 0.9966	8.2404
	S_4_ + P_0.5_	y = −8.2611x + 21.274	R^2^ = 0.9953	8.2611
	S_4_ + P_0.75_	y = −7.3347x + 18.959	R^2^ = 0.9999	7.3347
	S_4_ + P_1.0_	y = −7.4027x + 19.156	R^2^ = 0.9971	7.4027
	S_4_ + P_1.25_	y = −6.7658x + 17.571	R^2^ = 0.9964	6.7658
	S_4_ + P_1.5_	y = −6.6601x + 17.317	R^2^ = 0.9958	6.6601
	R_6_	y = −8.1127x + 20.876	R^2^ = 0.9853	8.1127
	R_4_	y = −9.0659x + 23.258	R^2^ = 0.9931	9.0659
	R_4_ + P_0.5_	y = −8.5545x + 22.001	R^2^ = 0.995	8.5545
	R_4_ + P_0.75_	y = −7.7112x + 19.888	R^2^ = 0.9998	7.7112
	R_4_ + P_1.0_	y = −7.2983x + 18.864	R^2^ = 0.9911	7.2983
	R_4_ + P_1.25_	y = −7.1512x + 18.519	R^2^ = 0.996	7.1512
	R_4_ + P_1.5_	y = −6.8034x + 17.647	R^2^ = 0.9953	6.8034
LH-90#	S_6_	y = −7.6056x + 19.617	R^2^ = 0.9894	7.6056
	S _4_	y = −8.7073x + 22.345	R^2^ = 0.9947	8.7073
	S_4_ + P_0.5_	y = −8.3059x + 21.359	R^2^ = 0.9998	8.3059
	S_4_ + P_0.75_	y = −7.9426x + 20.472	R^2^ = 1	7.9426
	S_4_ + P_1.0_	y = −8.017x + 20.684	R^2^ = 0.9974	8.017
	S_4_ + P_1.25_	y = −7.1883x + 18.625	R^2^ = 0.9988	7.1883
	S_4_ + P_1.5_	y = −6.8357x + 17.758	R^2^ = 0.9988	6.8357
	R_6_	y = −9.1534x + 23.457	R^2^ = 0.9995	9.1534
	R_4_	y = −9.6161x + 24.594	R^2^ = 0.9926	9.6161
	R_4_ + P_0.5_	y = −9.4301x + 24.171	R^2^ = 0.9962	9.4301
	R_4_ + P_0.75_	y = −8.4108x + 21.618	R^2^ = 1	8.4108
	R_4_ + P_1.0_	y = −8.6712x + 22.292	R^2^ = 0.9969	8.6712
	R_4_ + P_1.25_	y = −7.2864x + 18.836	R^2^ = 0.9986	7.2864
	R_4_ + P_1.5_	y = −6.9272x + 17.95	R^2^ = 0.9987	6.9272

**Table 6:** Performance changes of PPA–SBS modified asphalt before and after aging.

Asphalt Type	No	Retained Penetration Ratio (%)	Softening Point Increment (°C)	Mass Loss Rate (%)
DH-70#	S_6_	77.32	4.5	0.379
	S_4_	73.17	5.8	0.442
	S_4_ + P_0.5_	75.44	4.7	0.412
	S_4_ + P_0.75_	76.81	4.5	0.391
	S_4_ + P_1.0_	78.24	4.2	0.357
	S_4_ + P_1.25_	79.25	3.8	0.321
	S_4_ + P_1.5_	80.64	3.1	0.271
LH-90#	S_6_	71.25	5.1	0.418
	S_4_	66.15	6.9	0.533
	S_4_ + P_0.5_	69.03	5.4	0.435
	S_4_ + P_0.75_	71.39	5.2	0.401
	S_4_ + P_1.0_	73.51	4.8	0.379
	S_4_ + P_1.25_	75.28	4.0	0.314
	S_4_ + P_1.5_	76.78	3.3	0.258

**Table 7:** Performance changes of PPA–SBR modified asphalt before and after aging.

Asphalt Type	No	Retained Penetration Ratio (%)	Softening Point Increment (°C)	Mass Loss Rate (%)
DH-70#	R_6_	68.68	8.8	0.385
	R_4_	64.94	10.5	0.467
	R_4_ + P_0.5_	67.24	9.5	0.426
	R_4_ + P_0.75_	68.93	8.6	0.374
	R_4_ + P_1.0_	71.11	6.1	0.318
	R_4_ + P_1.25_	74.62	4.4	0.243
	R_4_ + P_1.5_	75.65	3.7	0.221
LH-90#	R_6_	61.26	10.4	0.422
	R_4_	57.19	12.0	0.486
	R_4_ + P_0.5_	60.24	10.2	0.452
	R_4_ + P_0.75_	61.87	8.9	0.371
	R_4_ + P_1.0_	65.02	6.6	0.309
	R_4_ + P_1.25_	68.75	4.3	0.227
	R_4_ + P_1.5_	71.96	3.3	0.172

## Data Availability

Data is contained within the article.
